# Structural Alterations of Human Serum Albumin Caused by Glycative and Oxidative Stressors Revealed by Circular Dichroism Analysis

**DOI:** 10.3390/ijms140610694

**Published:** 2013-05-23

**Authors:** Fiammetta Monacelli, Daniela Storace, Cristina D’Arrigo, Roberta Sanguineti, Roberta Borghi, Davide Pacini, Anna L. Furfaro, Maria A. Pronzato, Patrizio Odetti, Nicola Traverso

**Affiliations:** 1 Department of Internal Medicine, University of Genova, Genova 16132, Italy; E-Mails: fiammetta.monacelli@unige.it (F.M.); robertasanguineti@libero.it (R.S.); robertaborghi@yahoo.it (R.B.); diabolik451@gmail.com (D.P.); odetti@unige.it (P.O.); 2 Institute for Macromolecular Studies-ISMAC, National Research Council-CNR, Genova 16149, Italy; E-Mail: cdarrigo@ge.ismac.cnr.it; 3 Department of Experimental Medicine, Section of General Pathology, University of Genova, Genova 16132, Italy; E-Mails: annalisa.furfaro@unige.it (A.L.F.); maidep@unige.it (M.A.P.)

**Keywords:** ribose, ascorbic acid, pentosidine, fluorescence, circular dichroism

## Abstract

The aim of this work was to evaluate the ability of oxidative and glycative stressors to modify properties of human serum albumin (HSA) by analyzing markers of glycation (pentosidine) and oxidation (advanced oxidative protein products (AOPPs)) and assessing fluorescence and circular dichroism. HSA was incubated for up to 21 days with ribose, ascorbic acid (AA) and diethylenetriamine pentacetate (DTPA) in various combinations in order to evaluate influences of these substances on the structure of HSA. Ribose was included as a strong glycative molecule, AA as a modulator of oxidative stress, and DTPA as an inhibitor of metal-catalyzed oxidation. Ribose induced a significant increase in pentosidine levels. AA and DTPA prevented the accumulation of pentosidine, especially at later time points. Ribose induced a mild increase in AOPP formation, while AA was a strong inducer of AOPP formation. Ribose, in combination with AA, further increased the formation of AOPP. DTPA prevented the AA-induced generation of AOPP. Ribose was also a potent inducer of fluorescence at 335nm ex/385nm em, which is typical of pentosidine. AA and DTPA prevented this fluorescence. Circular dichroism showed complex results, in which AA and DTPA were strong modifiers of the percentages of the alpha-helical structure of HSA, while ribose affected the structure of HSA only at later time points.

## 1. Introduction

Over time, body proteins undergo non-enzymatic post-translational modifications, such as glycation and oxidation. These changes give rise to a multitude of different structures, many of which have not yet been identified. It is possible to mimic these modifications *in vitro* by incubating proteins with specific stressors.

Glycation is a reversible condensation of the aldehyde group of reducing sugars with a protein amino group to form a Schiff base, followed by an irreversible rearrangement to an Amadori intermediate. This intermediate undergoes cycles of condensations with additional amines, dehydrations, and oxidative fragmentations to yield heterogeneous chemical compounds collectively called advanced glycation end-products (AGEs) [1]. Oxidation can act directly through the action of reactive oxygen species (ROS) [2], indirectly through the generation of lipid peroxidation by-products (many of which are aldehydes) [3], or by specific redox-active molecules such as ascorbic acid (AA) [4]. The presence of heavy metal ions, such as iron or copper, facilitates oxidative damage through the catalysis of the Fenton reaction [5].

Many observations led to the conclusion that glycation and oxidation are strongly interconnected pathways. Glycation both enhances and is enhanced by oxidative steps, and a new term of “glycoxidation” has been coined to describe this quality [6].

Pentosidine is a highly fluorescent AGE compound that forms crosslinks between a molecule of arginine and a molecule of lysine, with an imidazo–pyridinium structure acting as a bridge. Its plasma levels increase with advanced age [7]. Pentosidine has been identified *in vivo* in skin collagen and plasma proteins of diabetic patients [8], in hemodialysis patients [9], in aged lenses [10], and in senile plaques of brain tissue from patients with Alzheimer’s disease [11]. Consequently, pentosidine is used as a fluorescent glycoxidation marker for AGEs that may reveal potential links with pathophysiological processes *in vivo* [12].

It has been suggested that protein glycation and the formation of AGEs are involved in structural and functional *in vivo* changes in proteins. However, very little information currently exists regarding the effects that these post-translational modifications exert on protein secondary or tertiary structure [13].

A previous study [14] of human serum albumin (HSA) suggested that glycation resulted in a conformational change, but the nature of this change was undetermined. Other studies have reported that glycation and the formation AGEs alter the helicity of proteins [15]. The extracellular matrix of proteins undergoes progressive changes during aging, which are characterized by decreased solubility, decreased proteolytic digestibility [16], and increased heat denaturation time [17].

The purpose of this study was to establish an *in vitro* model system to incubate HSA with ribose, a strong inducer of glycoxidation [18]. We also attempted to modulate the process of glycation through coincubation with other substances, including AA as an antioxidant [19] and diethylenetriamine pentacetate (DTPA) as a metal chelator.

Past reports on the impact of AA on *in vivo* glycation have been inconclusive. Both pro-oxidant and antioxidant properties have been attributed to AA, with a prevalence of antioxidant evidence. Some authors addressed ascorbate as a precursor of pentosidine [18,20]. In the present work, we consider it a modulator of oxidative stress, because it is able to generate a redox-active cycle between ascorbate and dehydroascorbate. Metal-catalyzed autoxidation of ascorbate may lead to the formation of ROS, including superoxide (O_2_^−^), hydrogen peroxide (H_2_O_2_), and hydroxyl radicals (OH^•^) [21]. DTPA is a powerful metal ion chelator that we used as an inhibitor of metal-catalyzed oxidation.

We measured pentosidine levels as a marker of glycoxidation and advanced oxidation protein product (AOPP) generation as a marker of oxidation. We measured fluorescence at 335 nm ex/385 nm em as a marker of pentosidine-like fluorescence and fluorescence at 370 nm excitation/440 nm emission as a generic marker of AGE formation. We also analyzed the protein conformation of HSA by circular dichroism (CD).

## 2. Results and Discussion

### 2.1. Pentosidine

**Figure 1:**
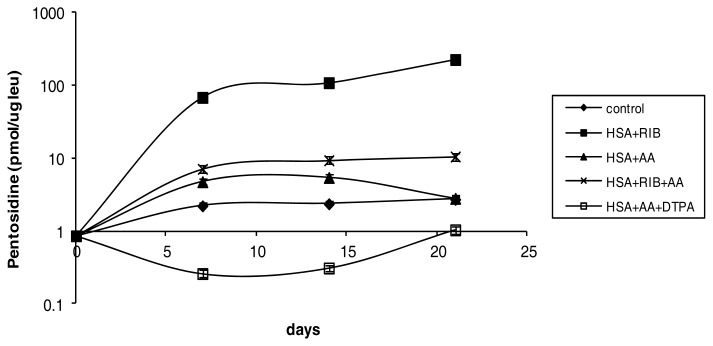
Effects of ribose, AA, AA + ribose, and AA + DTPA on pentosidine levels in HSA during 21 days of incubation.

Ribose was, as expected, a strong inducer of glycation. After 7 days of incubation with ribose, the pentosidine level was nearly 70 times the level in the control sample. After day 7, the increase was more modest, with a level 100 times the control after 14 days and 200 times the control after 21 days (*p* < 0.001 *vs.* respective control at each incubation time). In our experimental model, AA was a weaker glycation inducer (*p* > 0.05 *vs.* respective control), but the combination AA + ribose significantly counteracted the effects of ribose (AA + ribose *vs.* ribose *p* < 0.001 at any time). Under incubation with AA + ribose, pentosidine reached levels approximately 10 times the level in the control sample at 7, 14 and 21 days. Levels of pentosidine in the sample incubated with AA + DTPA were not significantly different from the levels in the control sample (Figure 1).

**Figure 2:**
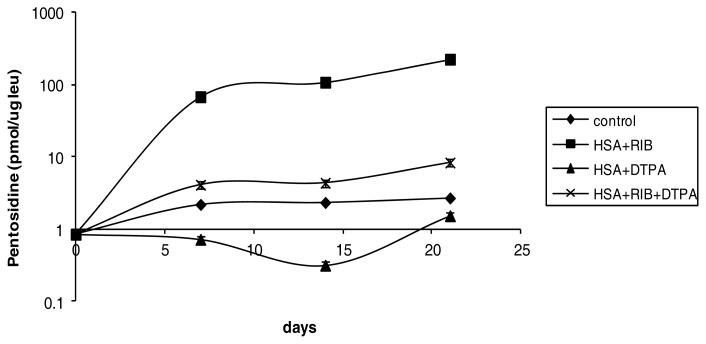
Effects of ribose, DTPA, and ribose + DTPA on pentosidine levels in HSA during 21 days of incubation.

DTPA was a stronger inhibitor of ribose-dependent glycation, exhibited by pentosidine levels that reached a maximum of 8 times the level in the control sample at 21 days of incubation (Figure 2) (*p* < 0.001 *vs.* respective ribose sample at each incubation time).

### 2.2. AOPP

**Figure 3:**
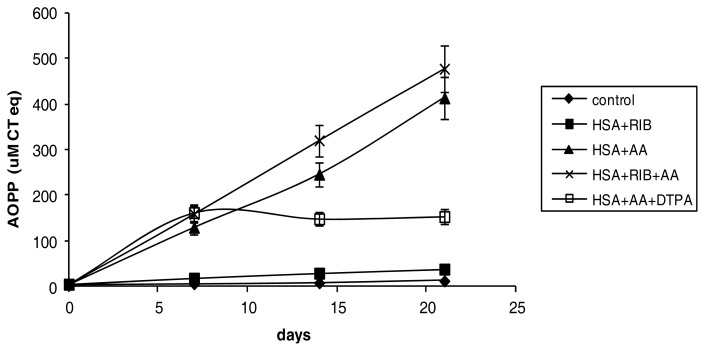
Effects of ribose, AA, ribose + AA, and AA + DTPA on AOPP formation in HSA during 21 days of incubation.

Ribose was less effective at inducing AOPP formation compared to AA. After 21 days of incubation with ribose, the level of AOPPs was approximately 10 times the level in the control sample (*p* < 0.001 *vs.* respective control). AA was the strongest inducer of AOPP formation in HSA. After 21 days of incubation, the AOPP level was approximately 75 times the level in the control sample (*p* < 0.001 at each incubation time). The combination of ribose + AA resulted in a steeper rise in AOPP, which may indicate that ribose enhanced the action of AA. After 21 days of incubation with ribose + AA, the level of AOPP was 85 times the level in the control sample (Figure 3), but the data did not reach significance relative to incubation with AA alone.

**Figure 4:**
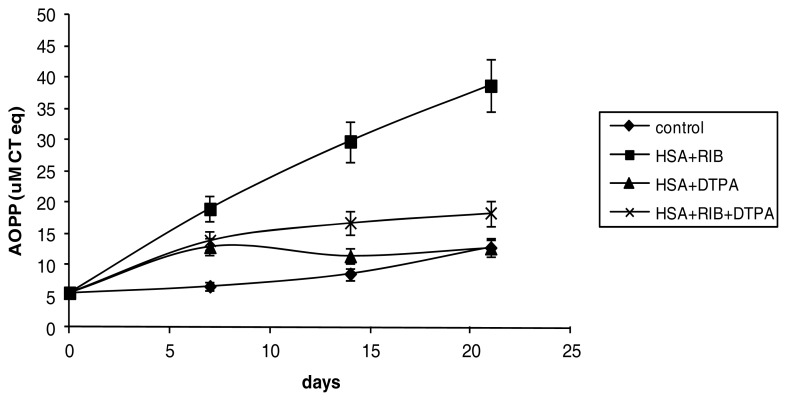
Effects of ribose, DTPA, and ribose + DTPA on AOPP formation in HSA during 21 days of incubation.

The combination ribose + DPTA significantly reduced the level of AOPP formation induced by ribose. After 21 days, the level of AOPP was 3 times the level in the control sample (*p* < 0.001) (Figure 4).

DTPA reduced the effects of AA on AOPP formation. After 7 days of incubation with DTPA, the level of AOPP was comparable to levels observed with AA incubation only. However, after day 7, the level of AOPP was relatively unchanged at approximately 27 times the level in the control sample (*p* < 0.001 AA *vs.* DTPA + AA at 14 and 21 days of incubation) (Figure 3).

### 2.3. Fluorescence

**Figure 5:**
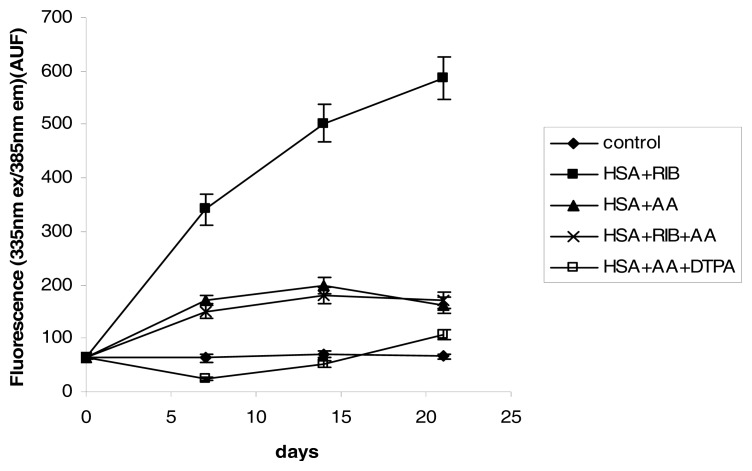
Effects of ribose, AA, ribose + AA, and AA + DTPA on 335/385 nm fluorescence of HSA during 21 days of incubation.

The fluorescence of pentosidine-like structures (335 nm ex/385 nm em) was similar to pentosidine, which was evaluated by HPLC. Ribose was a strong inducer of this fluorescence and reached a value of 9 times the control by 21 days of incubation (*p* < 0.001 at each incubation time). AA was a weaker inducer of fluorescence, but it counteracted the effects of ribose (ribose + AA *vs.* ribose *p* < 0.001 at each incubation time) (Figure 5).

**Figure 6:**
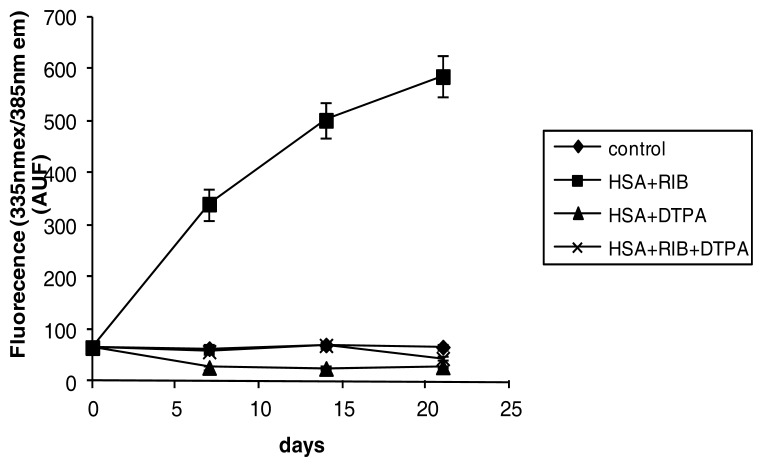
Effects of ribose, DTPA, and ribose + DTPA on 335/385nm fluorescence of HSA during 21 days of incubation.

DTPA virtually abolished the ribose-induced fluorescence of HSA (ribose + DTPA *vs.* ribose *p* < 0.001 at each incubation time) (Figure 6). Data observed with DTPA and ribose + DTPA were not significantly different from the control.

**Figure 7:**
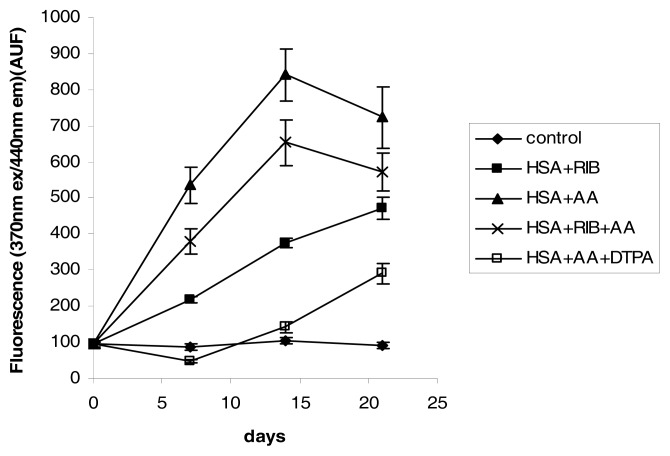
Effects of ribose, AA, ribose + AA, and AA + DTPA on 370/440 nm fluorescence of HSA during 21 days of incubation.

The fluorescence at 370/440 nm displayed different behavior than the fluorescence at 335/385 nm. Ribose was a strong inducer of fluorescence at 370/440 nm (ribose *vs.* control *p* < 0.001 at each incubation time), but AA was a stronger inducer than ribose (AA *vs.* control *p* < 0.001 at each incubation time). The fluorescence of the AA sample peaked at 14 days, with a value approximately 9 times the control. The ribose + AA combination provided intermediate results, indicating that ribose might inhibit AA-dependent fluorescence or AA might enhance ribose-dependent fluorescence (Figure 7).

**Figure 8:**
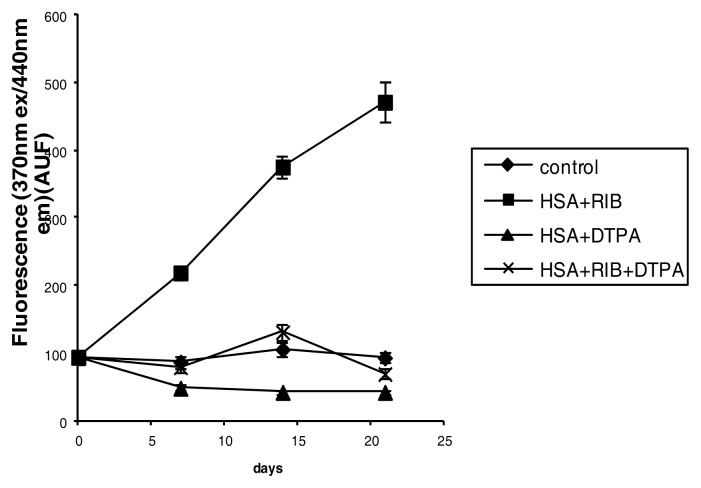
Effects of ribose, DTPA and ribose + DTPA on 370/440 fluorescence of HSA during 21 days of incubation.

DTPA abolished the ribose-induced fluorescence at 370/440 nm (ribose *vs.* DTPA *p* < 0.001 at each incubation time) (Figure 8).

DTPA significantly decreased the fluorescence at 370/440 nm compared to AA. The fluorescence peaked at approximately 3 times the control value after 21 days of incubation (AA *vs.* DTPA *p* < 0.001 at each incubation time) (Figure 7).

### 2.4. Circular Dichroism

**Figure 9:**
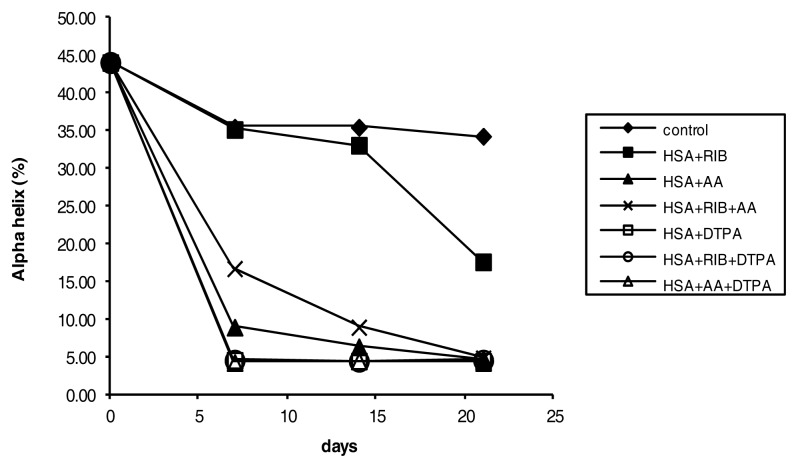
Percentage of alpha-helix conformation in HSA treated with ribose, AA, and DTPA and their combinations, as evaluated by CD.

**Figure 10:**
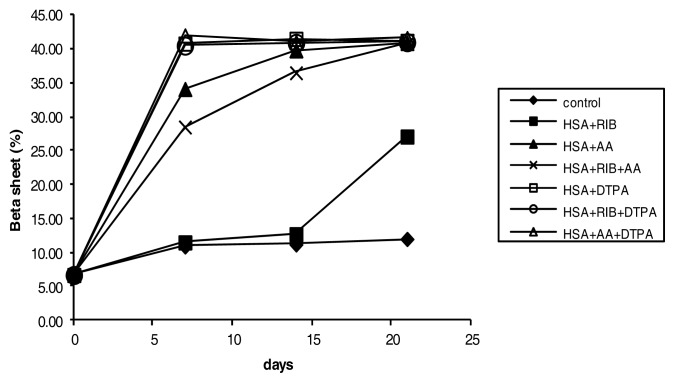
Percentage of beta-sheet conformation in HSA treated with ribose, AA, and DTPA and their combinations, as evaluated by CD.

The CD analysis indicated that ribose was a relatively weak and slow modifier of secondary protein structure. Ribose induced a significant reduction in the proportion of the alpha-helical structure of HSA (Figure 9), with a parallel increase in beta-sheet structures (Figure 10), but only after 21 days of incubation, compared to the respective controls. AA, ribose + AA, ribose + DTPA and AA + DTPA were strong modifiers of secondary protein structure (Figures 9 and 10). After 7 days of incubation, a significant decrease in the alpha-helix conformation was already evident (*p* < 0.05 for each incubation compared to the respective controls).

The ribose + DTPA and AA + DTPA combinations and, unexpectedly, DTPA alone were the most effective modifiers of secondary protein structure. When the alpha-helix structure decreased, the beta-sheet structure also increased.

No particular modifications were observed in the random-coil structure for any of the treatments evaluated (data not shown).

### 2.5. Discussion

In our model, ribose was very effective in generating pentosidine and inducing pentosidine-related fluorescence and Maillard-related fluorescence, in accordance with recent evidence from the literature [22]. CD indicated that ribose was a weak inducer of modifications of the secondary protein structure.

The behavior of AA was inconclusive [23]. On one hand, AA reduced ribose-induced pentosidine generation, but, on the other hand, it was an effective inducer of AOPP production. Ribose alone was not an effective inducer of AOPP formation, but it reinforced the effects of AA on AOPP formation. We believe that, in our model, AA may behave differently in the two biochemical backgrounds due to its intrinsic redox nature. It may act as a reductant (antioxidant) in the production of pentosidine [24] and as an oxidant in the production of AOPP. This double-action of AA is widely recognized [19,25]. AA also induced more Maillard-related fluorescence (370/440 nm) than ribose, indicating that AA may participate primarily in the generation of AGEs other than pentosidine. AA has also been suggested to inhibit *in vivo* glycation of albumin, hemoglobin and other proteins [26], while other authors have considered it a pentosidine precursor [18,20]. This, again, underlines the potential dual action of AA.

DTPA was effective at protecting HSA from both ribose-induced pentosidine formation and AOPP formation. DTPA also prevented pentosidine-like fluorescence (335/385 nm) and Maillard-related fluorescence (370/440 nm). These results underscore the critical role of heavy metal ions, such as copper and iron, in both glycation and oxidation of proteins. These observations also reinforce the idea of a strong interrelation between the two biochemical processes.

Rondeau *et al.* [27] reported that the glycation and glycoxidation processes of albumin induce several structural modifications, including an increase in the molecular weight of the protein. Non-enzymatic glycosylation of albumin *in vivo* occurs at multiple sites corresponding to arginine, lysine and cysteine residues. This structural modification is accompanied by a higher exposure of hydrophobic sites to the solvent. However, prolonged incubation with a carbohydrate, such as glucose, fructose or ribose, induces a transition in albumin from a helical to a beta-sheet structure, which is the basis of amyloid formation. By contrast, aggregate formation, which is induced by glycation, is not necessarily associated with secondary structure modification.

Short-term incubation (7 days) with d-ribose induces albumin to undergo rapid misfolding and form globular amyloid-like aggregations without any change in alpha-helix or beta-sheet proportions [22]. In addition, glycation of albumin results in an overall stabilization of both its tertiary and secondary structures. Indeed, glycation of human albumin changes the unfolding of the protein in the presence of a chemical denaturant reagent. The increase in protein stability attributed to glycation could have an impact on its half-life and may enhance the residence time of glycated albumin in the circulatory system [27].

CD is a powerful tool for investigating the structure of proteins [28]. Several authors have used CD to study conformational changes of proteins, even during glycation. CD provides signs of protein destabilization and alpha-helix reduction [29,30] However, some authors found that glycative agents interfered with glycation and glycation-induced conformational changes [31]. The type of protein also has a role in the conformational modifications induced by glycation, such as alpha- and gamma-crystallin [32]. Other authors indicated that ribosylation of proteins, known to be more rapid than glycation with other sugars, induced the formation of globulin-like aggregates, which were toxic in the neurotypic cell line SH-SY5Y [22]. Some scientists reported a glucose concentration-dependent change in CD spectra [33] and others observed partial denaturation and changes in the structural integrity of HSA using both low (1 mg/mL) and high (5 mg/mL) concentrations of glucose [34]. A change from alpha-helix to beta-sheets has been observed for the glycation of hemoglobin [35]. Other authors maintained that, in contrast to globular albumin, glycated albumin contains amino acid residues in a beta-sheet conformation, as measured with CD spectropolarimetry [36] Also, AA has been recognized as a possible inducer of secondary structure alterations evidenced in CD [37].

Mendez *et al.* [38] monitored the unfolding of HSA and glycated HSA (gHSA) subjected to guanidine hydrochloride (GndHCl) by using fluorescence and CD spectroscopy. The authors showed that glycation altered the local structure around Trp-214, but did not significantly impact the secondary structure of the proteins. This alteration translated into an overall change in the stability of gHSA compared to HSA. Both gHSA and HSA have nearly identical spectral shapes, indicating that glycation does not alter the overall alpha-helical structure of HSA. In the presence of 6 M GndHCl, both HSA and gHSA have identical CD spectra, indicating that both are equally unfolded. Although the difference in fluorescence between HSA and gHSA indicates that the local structure around Trp-214 is altered by glycation, the overall secondary structure is not significantly changed by the addition of the carbohydrate moieties.

Mohamadi-Nejad *et al.* [39] arrived at the same conclusions by studying the conformational changes that occurred during the incubation of HSA with different concentrations of glucose. A decrease in alpha-helical secondary structure accompanied lower stability at low glucose concentration (8.25 mM), while an increase in alpha-helices was associated with higher stability at higher glucose concentrations (16.5 and 27.5 mM). In the present study, we used 50 mM D-ribose, a high concentration that, according Mohamadi-Nejad, should increase the stability of the overall structure. Indeed, after 7 days, we did not observe any changes in secondary structure compared to the control samples. We only observed changes in alpha-helix and beta-sheet proportions of HSA after 21 days of incubation.

We observed significant changes in the proportion of alpha-helix and beta-sheet structures of HSA after 7 days of incubation with AA and DTPA. These results can be explained by the reduction of the protein compactness and consequent destabilization of the secondary structure owing to the exposure of hydrophobic sites to the solvent after a probable reduction of S–S bridges [40] from the antioxidants used in our experiment. Meucci *et al.* [41] investigated the action of a non-enzymatic metal-catalyzed oxidation system (ascorbate, oxygen, and trace metals) on HSA and their results were consistent with the role of ascorbate as a reducing agent that causes oxidative reactions via metal-mediated formation of H_2_O_2_, followed by the precipitation of traces of reduced metals, such iron and copper, in the Fenton reaction. The absorption spectra of native and modified HSA revealed that treated HSA is hyperchromic (10%–15%), indicating the partial disappearance of ordered structure. The hyperchromism is absent in the treated protein preincubated with AA in presence of DTPA, confirming their roles as antioxidants.

In our experimental model, CD indicated that ribose was a weak inducer of secondary structure modification from alpha-helices to beta-sheets, while AA and, unexpectedly, DTPA were more effective at inducing structural changes. It is possible that the maintenance of the alpha-helix structure may be a facilitating condition for pentosidine formation, which is evident in ribose incubations. Conversely, AA was a powerful inducer of the conformational transition from alpha-helices to beta-sheets, and it induced the formation of AOPP. Therefore, alpha-helix maintenance does not seem to be an essential characteristic for oxidative modification of proteins, at least in our experimental conditions. This may suggest that, in addition to the antioxidant effects, AA could inhibit pentosidine and pentosidine-like fluorescence generation by modifying protein structures, which, in turn, may become less susceptible to ribose activity. However, we have no direct evidence of this phenomenon.

This *in vitro* evidence is original and may offer an interesting field of speculation for further *in vivo* studies. The combination of AA and ribose significantly impacted glycation pathways and decreased AGEs formation, possibly due to a conformational secondary structure change in the protein. The data is innovative, since a great body of evidence acknowledges that ascorbylation of proteins drives oxidative and glycative pathways in humans, including the formation of pentosidine and vesperyline [42]. Our results suggest that high ribose concentration may be modulated by the pro-oxidative behavior of AA, which generates a conformational protein change that mitigates ribose activity and AGE formation.

Other data from the present study deserves further investigation. DTPA was a powerful modifier of secondary protein structural changes from alpha-helices to beta-sheets, indicating that heavy metal ions appear to be necessary for the maintenance of correct secondary protein structure. Moreover, if our hypothesis is correct, DTPA modification of the secondary protein structure might induce the formation of beta-sheet structure and, therefore, protect HSA from glycative damage.

If our model is correct, an *in vitro* human pathophysiological model is conceivable. Exposing proteins to long-term pathophysiological concentrations of AGE precursors, such as glucose-6-phosphate or metylglyoxal, could mimic chronic conditions like diabetes. This experimental framework could provide new insight into the Maillard reaction, which could be translated into the clinical field.

## 3. Materials and Methods

### 3.1. Glycation of HSA

Glycation of HSA was performed by incubating HSA (0.1 mM) in 50 mM D-ribose for 7, 14 and 21 days in the dark at 37 °C. In the same way, HSA was incubated with 20 mM AA, or both ribose and AA, at the same concentrations for the same incubation times. Similar experiments were performed with 10 mM DTPA and the combination of 50 mM ribose and 10 mM DTPA. Each mixture was solved in 2 mM HEPES (pH 7.2) containing 3 mM NaN_3_ and 2 mM phenylmetansulfonil fluoride. Incubation was conducted in sterile capped polystyrene test tubes in triplicate.

Control samples of HSA were prepared and treated similarly, except that no ribose, AA or DTPA was added to the mixture.

All samples were extensively dialyzed against 2 mmol/L HEPES (pH. 7.2) and filtered with 0.22 μm membranes (Millipore, Milan, Italy).

### 3.2. Determination of Pentosidine by HPLC

Pentosidine levels were determined by HPLC detection according to Odetti *et al.* [9]. A pentosidine standard was prepared as previously reported [18]. All samples were hydrolyzed with 6 M HCl for 20 h at 110 °C in borosilicate screw-capped tubes flushed with nitrogen. All hydrolyzed samples were dried in a SpeedVac^®^ Concentrator System (Savant Instruments Inc., Holbrook, NY), reconstituted in HPLC grade water containing 0.01M heptafluorobutyric acid (HFBA) as an ion-pairing agent, and filtered through a 0.45-μm pore diameter Ultrafree MC. Next, gHSA preparations were injected onto a Waters Xterra reverse-phase analytical column (C_18_ column 25 cm × 0.46 cm, 5 μm) connected to a Waters HPLC system with a curvilinear gradient program of 20%–40% phase B (methanol) for 30 min; solvent A was water. Both water and methanol contained 0.01 M HFBA. A synthetic pentosidine standard was injected at the beginning of every batch in order to quantify the pentosidine levels in the sample by peak area comparison. The amount of pentosidine was expressed as pmol per micrograms of leucine equivalent.

### 3.3. AOPP Assay

The levels of AOPPs, which are oxidation products with characteristic absorbance, were determined by spectrophotometric detection according to Witko-Sarsat [43]. The AOPP levels were measured by spectrophotometry on a microplate reader and were calibrated with chloramine-T (CT) solutions, which, in presence of potassium iodide, absorb at λ 340 nm. A 200 μL sample of HSA preparation (diluted 1/5 in PBS) was placed in the test wells of a 96-well microtiter plate; 20 μL of AA was added to each well. In standard wells, 10 μL of 1.16 M potassium iodide was added to 200 μL of CT solution (0–100 μM), followed by 20 μL of AA. The absorbance of the reaction mixture was immediately read at 340 nm against a blank containing 200 μL of PBS, 10 μL of potassium iodide, and 20 μL of AA. The AOPP concentrations were expressed as μmol/L of CT equivalents.

### 3.4. Fluorescence Measurements

Fluorescence intensity was determined with an L55B Perkin-Elmer (Verkauf, Munchen, GmBH) spectrophotofluorometer at 440 nm upon excitation and 370 nm emission for Maillard product-related fluorescence [44] and 335 nm excitation and 385 nm emission for pentosidine-like products [9]. The absorbance of a protein control solution was determined in order to rule out any possible interference on fluorescence evaluation. The absorbance of the protein solution at the wavelengths used for fluorescence excitation never exceeded 0.1. The intensity of fluorescence was expressed in arbitrary units of fluorescence (AUF) per milligram of leucine equivalent.

### 3.5. Amino Acid Estimation

Amino acid concentrations were estimated in acid hydrolysate with the ninhydrin reaction, using L-leucine as a standard. The leucine levels in the samples were estimated by a photometric method as previously described [45], with some modifications. Briefly, the gHSA samples were hydrolyzed with 6 M HCl for 20 h at 110 °C in borosilicate screw-capped tubes that had been flushed with nitrogen. A portion of each of the above acid hydrolysates were evaluated to estimate the α-amino nitrogen in the fractions by reaction with the modified ninhydrin reagent of Moore and Stein [45]. The hydrolysate HSA samples were then diluted as appropriate and mixed with ninhydrin solution in 4 × 0.5-inch tubes with metal caps and placed in a boiling water bath for 15 min. The diluent (equal volumes of ethanol and distilled water) was added and left at room temperature for 15 min. The optical density at 570 nm was read and the amino acid concentration was calculated with a standard response curve prepared with known graded concentrations of leucine. The amount of material present in each fraction was calculated in terms of leucine equivalent by reference to this standard curve.

### 3.6. CD Measurements

The concentration of the HSA in 2 mM HEPES was evaluated before each measurement with a Cary UV-Vis spectropolarimeter. The spectropolarimeter was calibrated with an aqueous solution of (1S)-(+)-10-camphor sulfonic acid at 290.5 nm in accordance with the instrument manual. The spectrum at each time was obtained by averaging three scans from 195 to 250 nm at a rate of 20 nm·min^−1^ with a step resolution of 0.2 nm, a time constant of four seconds, and a bandwidth of 2.0 nm. A thermostated 0.1 cm path-length cell was used. All measurements were taken at 20 °C. The data were expressed in term of [Θ], the mean residue ellipticity expressed in units of degrees centimeter squared per decimole of residue. All the spectra were corrected by subtracting the appropriate buffer background and normalized to mean residue ellipticity. Deconvolution of the resulting spectra was achieved using the CONTINLL program to determine the relative amounts of the alpha-helix and beta-sheet secondary structure components. Far UV CD spectra were recorded on a Jasco J-500A spectropolarimeter equipped with a Jasco IF-500-2 data processor [46].

### 3.7. Statistical Analysis

The results represent the mean values of at least three independent experiments performed under the same conditions. Results were expressed as mean ± SD. The statistical significance of experimental data was evaluated by parametric one-way ANOVA, followed by Bonferroni post-test. A *p*-value < 0.05 was considered significant. Analyses were conducted with the GraphPad Prism 4.0 software package (GraphPad Software, San Diego, CA, USA).

## 4. Conclusions

Analyses of HSA incubated with ribose, AA and DTPA in various combinations indicate that the conformation of proteins could affect their susceptibility to glycation and oxidation.

## Abbreviations

HSA: human serum albumin
DTPA: diethylenetriamine pentacetate
AOPP: advanced oxidation protein product
AGE: advanced glycation end-product
HFBA: heptafluorobutyric acid
CT: chloramine-T
AUF: arbitrary units of fluorescence
CD: circular dichroism
AA: ascorbic acid
